# ‘You must carry your wheelchair’ – barriers to accessing healthcare in a South African rural area

**DOI:** 10.3402/gha.v8.29003

**Published:** 2015-10-01

**Authors:** Richard Vergunst, Leslie Swartz, Gubela Mji, Malcolm MacLachlan, Hasheem Mannan

**Affiliations:** 1 Alan J Flisher Centre for Public Mental Health, Department of Psychology, Stellenbosch University, Stellenbosch, South Africa; 2 Centre for Rehabilitation Studies, Stellenbosch University, Stellenbosch, South Africa; 3 Centre for Global Health, School of Psychology, Trinity College, Dublin, Ireland; 4 School of Nursing, Midwifery & Health Systems, Health Sciences Centre, University College Dublin, Dublin, Ireland

**Keywords:** disability, rural, access, health, barriers

## Abstract

**Background:**

There is international evidence that people with disabilities face barriers when accessing primary healthcare services and that there is inadequate information about effective interventions that work to improve the lives of people with disabilities, especially in low-income and middle-income countries. Poor rural residents generally experience barriers to accessing primary healthcare, and these problems are further exacerbated for people with disabilities.

**Objective:**

In this study, we explore the challenges faced by people with disabilities in accessing healthcare in Madwaleni, a poor rural Xhosa community in South Africa.

**Design:**

Purposive sampling was done with 26 participants, using semi-structured interviews and content analysis to identify major themes.

**Results:**

This study showed a number of barriers to healthcare for people with disabilities. These included practical barriers, including geographical and staffing issues, and attitudinal barriers.

**Conclusions:**

It is suggested that although there are practical barriers that need to be addressed, attitudinal barriers could potentially be addressed more easily and cost effectively.

It is well established that access to healthcare may be compromised in low-income and middle-income countries (1). People with disabilities are more likely to experience access barriers in a range of contexts than the general population (2–15). Attitudinal barriers, in particular, hinder access to healthcare for people with disabilities (16, 17). Despite this, there remains a paucity of specific information on healthcare access for people with disabilities; such information is essential for the development of effective interventions that work to improve the lives of people with disabilities. Indeed, there has recently been a call for ‘urgent attention to the issue of access to appropriate healthcare for people with disabilities especially in low-income and middle-income countries’ (18).

People with disabilities experience unmet healthcare needs which lead to health disparities (19). The issue that people with disabilities should be treated the same as persons with no disabilities has been ratified by the Convention on the Rights of People with Disabilities (Article 25d), United Nations 2006 (20). Research contributing to improvement of health of people with disabilities needs to be prioritized.

Access to healthcare is more difficult in rural than in urban areas, and the difficulties with access are exacerbated for people with disabilities living in poverty in rural contexts. Poor people with disabilities who live in poor rural societies experience unique problems in accessing health services (21, 22). In addition, there is a higher prevalence of disability in rural areas compared with urban areas (23). However, disability and access to healthcare among the poor rural populations has received little attention. There are scarce data on their health needs (22). Meeting the healthcare needs of rural residents with disabilities, especially those living in poverty, will require interventions beyond healthcare, involving access more broadly, including, access to safe affordable transport (24, 25). These barriers are experienced across a range of age groups of people with disabilities (from children through to the elderly), and also for a range of services (from general primary healthcare to specialized HIV and mental health services) (26). In this study, we explore the challenges faced by people with disabilities in accessing healthcare in Madwaleni, a poor rural Xhosa community in South Africa.

The concept of rural health has developed considerably from earlier, more limited, understandings of rural health referring only to medical practice in rural areas (27, 28). To understand rural health, it is important to understand a minimum of three primary domains which have historically been central to the definition of what is rural. These are the ecological, the occupational, and the sociocultural components (29). The ecological component refers to the spatial apportionment of the population. This is conventionally employed to signify a delimited geographical area characterized by a population that is small, relatively sparse, and isolated, to varying degrees, from metropolitan hubs. The occupational dimension is construed as a well-defined, rather narrow, attribute of an aggregate of individuals who derive their livelihood from agricultural production, or perhaps more broadly from employment in extractive industries, such as mining, fishing, forestry, and so on. The sociocultural dimension of rurality is the most complex and least well-articulated, but generally refers to value structures or shared ideals that serve as the fundamental underpinnings of patterned interactions.

Concern over the availability of health services in rural areas has existed for decades (30). Rural residents have fewer options in seeking and receiving healthcare services (31) and ‘often confront significant barriers when seeking healthcare’ in the United States (24).

It is not sufficient, in itself, that a system of primary care be available in rural areas. The services must also be accessible (31). Accessibility here refers to the patient's ability to enter the primary care system without financial, geographic or organizational barriers that unnecessarily restrict entry into the system. Terrain, travel distances, population density, and transportation are all important factors in enabling a rural community to provide, and access, services (32). Access to rural primary care is significantly affected by the number and mix of providers. The shortage of healthcare professionals in rural communities is a global problem that poses a serious challenge to equitable healthcare delivery (33), issues which are exacerbated in the context of disability.

Rural residents are more likely to delay accessing care due to financial barriers than their urban counterparts. Travel times to doctors are on average longer for rural residents than for their urban counterparts. Overall, rural residents report higher levels of chronic conditions but they do not visit the doctor more frequently than do urban residents. The rural environment presents extraordinary threats to health (34), with sparse healthcare options in rural communities exacerbating difficulties (24). Healthcare providers face challenges in rural areas and this can create barriers to care (35). In this article, we examine access issues for people with disabilities in rural South Africa. In addition to sharing similar features with other low-income and middle-income countries regarding healthcare access, South Africa faces particular challenges.

## Rural health in South Africa

Rural practice, like virtually every other activity in South Africa, has been deeply shaped and impacted on by the political situation in the country under apartheid (36). Rural health in South Africa is synonymous with the health of the populace in the deliberately underdeveloped areas of the country, inhabited largely by black communities. Since the advent of democracy in 1994, there have been deliberate policies which attempt to redress the imbalances of the past in healthcare; the implementation of these policies remains a work in progress (36).

In South Africa, 52% of the total population and 75% of poor South Africans live in rural areas (37). Populations living in rural areas are largely the very young and the elderly, with the employable men and women finding work in the cities. This has serious consequences for the health of rural families. Another major factor influencing rural health is that of income and poverty.

South African society is a society in transition and this is reflected in its morbidity, mortality, and disability profiles (36). The health status of rural people in South Africa is similar to that of people in many developing nations around the world. The diseases of poverty are common, including chronic disability. Access to healthcare for rural people is difficult, as has been mentioned. There is also a plurality of health systems, with some rural people making use of indigenous and faith-based health systems. Public healthcare in rural areas has been rendered through a system of rural hospitals and clinics, many of which were built and operated as mission hospitals until the 1970s. Thereafter, most of these hospitals and clinics were controlled by the apartheid government in an effort to centralize planning. These same hospitals now form the infrastructure for the new National Health System, the aim of which is to decentralize to a district-based health system. The infrastructure and facilities available in rural hospitals, by South African government hospital standards, are relatively good, although diagnostic services are limited. Most rural hospitals offer comprehensive services and are staffed by nurses, allied health professionals, and by generalist doctors who have done their medical studies abroad.

The 2011 South Africa census came up with a disability prevalence of 7.5% among South Africans aged 5 years and older. The prevalence of disability in the Eastern Cape province of South Africa is 9.6% (38), whereas disability statistics in the Amatole district of the Eastern Cape (wherein the study was conducted) are unknown (21).

In summary, we now know that people with disabilities have poorer access to healthcare compared with people without disabilities, that all people in rural areas have more problems in terms of access to healthcare generally, and that people with disabilities in rural areas face specific barriers to access. In the context of a larger international study looking at access to healthcare for people with disabilities in poverty-stricken areas in Africa, we report here on qualitative information gleaned in a deeply rural, impoverished area in South Africa. These stories of the experiences of disability in a rural area form an important first step in our understanding of the issues at stake; issues which will be explored more extensively in a larger quantitative phase of the study.

## Methods

A purposive sampling process was used. Known healthcare providers and community leaders were included, as well as health service users (who were people with disabilities). Semi-structured interviews, looking at disability and access to healthcare, were carried out. A total of 26 interviews were completed. See Table 1 for sample characteristics.

**Table 1:** Sample characteristics

Sex	
Female	18
Male	8
Age	
5–17 years (financially dependent)	1
18–60 years (economically active)	16
61+ (potential pension)	9
Participant type	
Users	9
	5 with physical impairments
	2 with psychosocial impairments
	1 with sensory impairment
	1 with cognitive and physical impairment
Providers (e.g. medical staff)	9
Community Members (e.g. chiefs and traditional leaders)	8

These interviews were carried out at the Madwaleni Hospital, health centers, and at peoples’ homes by two of the authors. Interviews were on average 1 h in duration. A translator was used when interviews were carried out in Xhosa. The translator was present in all interviews when needed. Interviews were recorded and transcribed in English. The ATLAS TI program was used to analyze the transcribed data. Data were analyzed using thematic content analysis. Factors relating to disability and healthcare access were identified and coded. Codes were then grouped into themes on which the results are based.

Ethical clearance was obtained from the Eastern Cape Department of Health as well as from the University of Stellenbosch (REF N09/10/270).

### The setting

Madwaleni (one of 17 study sites of a large international research study (EquitAble) looking at disability and access to healthcare) is a rural area on the Wild Coast in South Africa's Eastern Cape Province. The area is characterized by rugged hills, rivers, forests, unpaved gravel roads, free running animals, and grass-thatched huts scattered sporadically over the hills. There is a scarcity of sewage systems, running water, and electricity supply to the general Madwaleni community, as these are limited to the hospital and the local hotels.

The Madwaleni Hospital is situated on the rolling hills of the Elliotdale district in Mbhashe Local Service Area. The area is also served by eight clinics: Hobeni, Nkanya, Bomvana, Molitafa, Soga, Xhora, Mqhele, and Mkhatazo. There are two major rivers and several other tributaries and streams. The major rivers are the Mbashe River and the Xora River. Madwaleni Hospital and the clinics Hobeni, Nkanya, Bomvana, Molitafa, and Soga are situated between the two major rivers, whereas the clinics Xhora, Mqhele, and Mkhatazo are situated on the banks of the Xora River. The medical and rehabilitation staff at the hospital is Caucasian, whereas the nursing staff are Madwalenian/Amabovane.

The Madwaleni area has a population of about 260,000. Along with the hospital and eight health centers, there are also many primary and secondary schools, which are well attended. There is also an OVC (Orphans and Vulnerable Children) center in the community.

## Results

There were numerous barriers to accessing healthcare for people with disabilities in Madwaleni.

### Geographical barriers

In terms of geography, getting to the hospital was ‘another story’ (Female community member, CM), with some patients having to relocate from their homes to the home of another member of their family who ‘lives close to the road’ (Female user). As one respondent put it:

… main barrier was geography and the distances between health centers - not strategically placed but rather at roads. People in the hills and valleys are often not accessed ….

The terrain was also an issue in that the wheelchair users had to deal with ‘mud’ (Female user), ‘gravel’ (Male user), and ‘uneven roads’ (Female user) and found it to be ‘quite taxing on their family and on them to push up a hill’ (Male user). ‘I don't have enough strength to actually push it myself’ (Female user). There are ‘footpaths and then … gravel road … hilly’ (Female CM) on which one has to manage a wheelchair. There are ‘hills and valleys’ (Female CM) and ‘two rivers and a forest’ (Female CM) making it ‘very difficult to access the nearest health clinic’ (Female CM). In an extreme situation, the community ‘have lost people to drowning when trying to access health care’ (Female CM).

Distances were also seen as a ‘main’ (Female provider) barrier. People had to ‘travel a distance’ (Female provider), as healthcare services were ‘situated so far’ (Female provider), and it took ‘so long’ (Female CM) as it was ‘a long trek (journey)’ (Male user). To summarize, ‘access to hospital in Madwaleni … not satisfied … hospital should come to the people’ (Male CM). The issue of distance has implications for health behavior:

Because of that, everything is sort of set back because they don't pick up signs and symptoms early enough. And because of the roles they gave to this hospital, it takes a long time. That also could be a factor in patients not wanting to come, because it's so long and because it's situated so far. You see in houses that they don't feel the patients can walk up. So if I lived in a place that was an hour walk from a hospital, and I just did not feel well that day, I wouldn't want to walk up the hill to the hospital …. (Male user)

The suspension of a previous mobile service also had implications:

He recalled that there was a mobile health unit which used to visit the villages in the community and it meant that services reached the community rather than the community making the long trek (journey) to the hospitals. These visits also facilitated health promotion, and since the stoppage of this aspect of the hospital's work, the issue of transportation has compounded the health of the Amabovanes and a deterioration of health promotion activities. (Male CM)

### Transport barriers

Transport was found to be ‘expensive’ (Female CM), and there is an ‘issue with taxis’ (Female user). Privately owned minibus taxis are the only form of public transport, with the result that transport is a ‘huge undertaking’ (Female user). Emergency transport is a particular problem, with people having to ‘arrange transportation the night before’ (Female user) and ‘having to get up early’ (Female user). Transport barriers experienced by users were described as follows:

… what stands out is transport, especially because her chair is a bit bulky and … taxi drivers put an extra chair because they want to fit in as many people as possible. So for her it is transport …. (Female user)

Transport is a huge problem. They are always told, you have to carry your sticks, and if it is one using a wheelchair, you must carry your wheelchair. So it's all this complaining around us. He says that for his leg it is better if he's sitting in the front seat, and not all cars can. Sometimes, when the cars pass, the front seat is already full and they pass him. So transport is an issue …. (Female user)

But now on rainy days, just going to hospital with my wheelchair, I pick up so much mud that it becomes difficult to get to work. The problem is, the way the roads are and the chair, I don't have enough strength to actually push it myself. And that is challenging, and if they were motorized it would take away that problem of needing some form of assistance …. (Female user)

### Organizational barriers

There were many organizational barriers presented. ‘Buildings are selfish in terms of their design’ (Male user), ‘shortage of staff’ (Female provider), ‘waiting period is over half a day on average’ (Female user), ‘poor management’ (Female provider), ‘it's difficult to get resources’ (Female provider), ‘we are short on stock’ (Female provider), ‘we have no crutches available’ (Female provider), and ‘shortage of supplies’ (Female provider). As one female provider sums up:

I enjoy working here. It's quite challenging because it is so far away from home. But it becomes problematic because it's difficult to get resources. I mean, I have been here since the beginning of the year and we have never received a lot of equipment. So it's difficult when you don't have enough resources. I feel like I don't give my patients effective treatment.

### Attitudinal barriers

Attitudinal barriers were also highlighted. For example, the ‘stigma of being disabled’ (Female user) and therefore they do ‘not access’ (Male user) the health clinics or hospital because of stigma. The fear of meeting people itself is a barrier because of the ‘stigma’ (Female CM). ‘If I'm disabled, I'm not going to meet with other people’ (Female user). Barriers were also due to ones ‘own fears and assumptions about nurses’ … view on disability (Female CM). As one female provider put it:

No. It is the stigma of being disabled …. If one is disabled and in a wheelchair, and I'm having incontinence and … it is embarrassing to the community at large because of my diagnosis. And if I'm disabled and I'm limping, seeing the tears and everything, I'm embarrassed to meet a group of people. I think it's one of the barriers to meet the other people. Because for the disabled one it's difficult to come here – she'll send someone. If she is taking treatment here, she sends someone to go to the clinic to fetch her treatment. It's us who send the community workers to see that person and what is her problem or his problem. One day, I'm asking a blind and deaf somebody, but he's working. He's blind and deaf, and I asked him one day to bring the person here to fetch his treatment by himself – and he's coming …. Yes, it's a barrier because of the stigma. If I'm disabled, I'm not going to meet with other people.

## Discussion

### Understanding the combination of barriers

It is clear that the barriers mentioned in the literature in terms of access to healthcare do apply to the situation in Madwaleni. More interesting than simply listing these barriers, however, is an examination of the interactions among these barriers and how they paint a more complex picture and give a full account of the dynamics that are happening when a person with disabilities has to access healthcare in Madwaleni.

The barriers to receiving healthcare in Madwaleni often start before the person leaves his or her home on what can often turn out to be a very difficult journey. A person with disabilities may have fears and assumptions about how nurses would react to their disability. These perceived staff attitudes toward disability often constitute a barrier in itself. Furthermore, there are times when the stigma held by the community of being a person with a disability hinders, and in extreme cases, prevents the person from even attempting to access healthcare – it may be less stressful to remain at home.

Attitudinal barriers within healthcare are a common experience for persons with disabilities. Attitudes of healthcare workers toward persons with disabilities were identified as a barrier in a rural study in South Africa (39). Negative attitudes toward individuals with psychosocial disabilities have been cited as an important barrier, leading to poor communication with primary care providers and the provision of less than adequate care (40). A study by Tracy and McDonald (41) found that people with intellectual disabilities continue to experience ‘multiple, complex and interrelated barriers’ (p. 24) to healthcare including attitudinal barriers.

In general, people with disabilities had to travel very long distances, which often involve very rough and treacherous terrains, to receive healthcare in Madwaleni. Beyond the long and difficult distances, the mode of transport as such is a complex issue in itself. People with disabilities have to generally rely on the main mode of transport in the area, namely taxis, which are sporadic. These taxis are often jam-packed, and they ply on dusty, gravel roads, making the journey unpleasant. For people with disabilities, access to health centers becomes difficult as they have to travel in these overcrowded taxis, and all the more difficult when they have to carry their wheel chair or crutches with them. But the practicalities of distance and transport are not the only barriers. Taxi drivers also complain of having to transport a person with disabilities along with a bulky wheelchair in a crowded taxi. The title of this article, ‘You Must Carry Your Wheelchair’, reflects both the absurdity of the situation and also the depth of stigmatizing attitudes which may exist under general conditions of poverty and exclusion. In this way, transport is not merely a technical issue but one with attitudinal factors, and these factors in themselves may relate to the broader context of poverty.

Compounding these difficulties of distance and transport to the hospital, the patients then encounter physical barriers in the form of poorly designed buildings. The hospital layout is perceived by certain CMs as not always being wheelchair friendly. Furthermore, the healthcare system has its own issues and factors that make healthcare more inaccessible. There is generally a shortage of staff at the hospital. This results in very long waiting times with an average waiting time of half a day. This, coupled with the time taken to travel the long distance, makes it an inordinately long journey before one receives healthcare. Also, a lack of management at the hospital and general lack of support from government makes it difficult to get resources essential for the adequate healthcare of people with disabilities. The unreliable supply of these essential resources to the hospital and clinics means that a person with a disability often has to make the long and difficult journey more than once to receive complete treatment.

### Some implications

This was the first known study in South Africa which looked at access to healthcare for people with disabilities in a rural community. This study supports international evidence that people with disabilities face barriers when accessing healthcare services (18) and supports studies looking at rural populations of people with disabilities and their access to healthcare (26).

Geographical barriers, with special reference to transport, terrain, and distance, were highlighted as significant barriers. This is supported by Paez et al. (42) who state that geographical access to healthcare facilities is known to influence health service usage, while Peters et al. (43) state that geographic access is an important part of assessing healthcare in low-income and middle-income countries. Terrain, travel distances, population density, and transportation are all important factors in the capacity of a rural community to provide services (32).

The location and distribution of healthcare services and the quality of transportation in terms of geography has received increased attention (42). Accessibility, defined as the travel impedance between patient location and the locations where care is delivered, come to the fore as an approach to understanding the geographical dimensions of healthcare (44). Until recently, relatively little was known about the geographical accessibility to healthcare (42).

An inverse relationship between distance or travel time to health facilities and use of health services has been demonstrated as an important barrier to access (45). Morrison et al. (5) found ‘multiple barriers’ that limit care for people with disabilities, including transportation.

Distance has been shown to matter in previous research (42). Studies in developing countries have presented strong evidence that the physical proximity of health services can play an important role in the use of primary healthcare (46, 47). However, distance as a barrier in itself does not fully explain accessibility, since transportation and mobility factors are also influential (36). In particular, while the individual and environmental factors that may pose barriers to healthcare have been independently studied, there has been only limited research into the way the individual and his or her environment may interact to influence accessibility levels. This interaction becomes particularly important in people with disabilities. This was seen in our results when we explored the interaction of barriers to healthcare in Madwaleni.

It is argued that measuring access to health services in developing countries remains imprecise and relies mostly on asking patients about the time and distance they traveled (48). Experts suggest that the use of geographic information system (GIS) – a system designed to capture, store, manipulate, analyze, manage, and present spatial and geographical data is more reliable and valid. According to a study measuring geographical access to health services, the use of self-reported data is not reliable because it is difficult for patients to remember the distance they have traveled or the time of the journey (49). These factors need to be considered when interpreting the results of this study as self-reported methodology was used.

### Limitations of study

This study used a small sample from a specific context and hence we need to be very careful in making any form of generalizations. Language barriers in terms of collecting and then analyzing the data may have occurred, resulting in the loss of potentially more in-depth experiences of the subjects. The lack of inclusion of all types of disabilities in the sample could also be considered a shortcoming in terms of looking at access and disability.

## Conclusions

In considering the barriers which exist for people with disabilities in accessing healthcare, many concrete issues can be identified, namely transport systems, distances traveled, road conditions, and staff provisioning. Overcoming these barriers would, due to their nature, incur substantial costs. However, underlying attitudinal barriers are equally important and could potentially be addressed without comparable expenditure. This is not to underestimate the complexity and challenge involved in addressing these attitudinal issues. Stigma barriers are potentially disabling in themselves, and may for instance mean that people with disabilities do not even get out of their home to access health services. In the case of our sample, wide geographical distribution and social disempowerment combine to marginalize people with disabilities. This is perhaps symbolized in the absurdity of the statement, ‘you must carry your wheelchair’, exemplifying both the denial of physical difficulties, and the socially hostile circumstances under which some people with disabilities live. This hostility may be rooted in South Africa's history of holding difference ‘apart’ (apartheid), and in its struggle to acknowledge the value of social inclusion in resource-poor areas. A stronger societal orientation toward, for instance, social justice, poverty relief, and employment may provide a platform for improved attitudes to people with disabilities. The issue of access to healthcare for rural South African people with disabilities is not therefore simply a disability issue, but a broader human rights issue, for all.
